# 

**Published:** 1896-12

**Authors:** 


Vol XVII. DECEMBER, 1896. No. 12.

PUBLISHED MONTHLY AT $3 A YEAR. SINGLE COPIES, 30 CENTS.

LIBRARY Indexed S.G.O.

### THE JOURNAL OF COMPARATIVE MEDICINE AND VETERINARY ARCHIVES

EDITED AND PUBLISHED BY
RUSH SHIPPEN HUIDEKOPER, Veterinarian (Alfort),
W. HORACE HOSKINS, D.V.S.,
H. D. GILL, V.S.

### UNITED STATES VETERINARY MEDICAL ASSOCIATION.

**Proceedings of the Thirty-third Annual Meeting held at Buffalo, N. Y., September, 1896. (Continued.)**

Report of the Committee on Diseases—*concluded*
Report of the Committee on Intelligence and Education . . . . . . 71
The Presidential Address . . . . . . . . . . . . . . . . 83
The Relations of Horseshoers to Veterinarians and Veterinary Colleges. By H. D. GILL, V.S. . . . . . . . . . . . . . . . . 90

ALL COMMUNICATIONS AND PAYMENTS SHOULD BE ADDRESSED TO
**Dr. W. Horace Hoskins, Managing Editor, 3452 Ludlow St., Philadelphia, Pa.**
Copies may be had on order of all news-dealers. In London of Baillière, Tindall & Cox.

